# Prevalence, knowledge, attitudes, and practices related to e-cigarette use among students in Kuwait: a cross-sectional study

**DOI:** 10.3389/fpubh.2026.1947592

**Published:** 2026-10-08

**Authors:** Fajer AlBarrak, Saleh Alsarhan, Maryam AlHamad, Qout Al-Mekhled, Jenan AlMatrouk, Sultan E. Alsalahi, Abdulaziz Alhenaidi

**Affiliations:** 1 Ministry of Health, Kuwait City, Kuwait; 2 Department of Health Policy and Management, College of Public Health, Kuwait University, Kuwait City, Kuwait; 3 Department of Public Health Practice, College of Public Health, Kuwait University, Kuwait City, Kuwait; 4 Department of Social and Behavioral Sciences, College of Public Health, Kuwait University, Kuwait City, Kuwait

**Keywords:** attitude, e-cigarettes, knowledge, Kuwait, practice, students

## Abstract

**Background:**

The global increase in electronic cigarette (e-cigarette) use represents a significant public health concern, especially among young adults in high-income countries and the Middle East. Although e-cigarette use is rising in Kuwait, comprehensive data regarding the knowledge, attitudes, and practices of university students are limited.

**Objective:**

This study assessed the prevalence of e-cigarette use and identified demographic, social, and cognitive factors associated with its use among students in Kuwait.

**Methods:**

A cross-sectional study was conducted from November 2025 to February 2026 using a stratified one-stage cluster sampling method across public, private, and Public Authority for Applied Education and Training (PAAET) institutions. A self-administered questionnaire was distributed to 550 students (57.3% male; mean age 20.89 years) to assess knowledge, attitudes, and patterns of e-cigarette use.

**Results:**

Approximately 50.4% of participants reported ever using e-cigarettes, and 22.5% reported current daily use. Overall knowledge (42%) and attitude scores (47%) were low, reflecting limited awareness of risks and generally non-accepting attitudes. Ever-users demonstrated significantly higher knowledge and more permissive attitudes compared to non-users. Multivariable logistic regression identified having friends who use e-cigarettes as the strongest predictor of use (AOR = 4.53), followed by male gender (AOR = 3.70) and enrollment in PAAET or private universities.

**Conclusion:**

The prevalence of e-cigarette use among Kuwaiti students is high relative to regional averages, primarily influenced by social and demographic factors. Public health interventions should extend beyond basic education to address the “knowledge-attitude paradox” and promote stricter regulations, including taxation and retail controls.

## Introduction

The use of electronic cigarettes (e-cigarettes) has increased substantially worldwide over the past decade, particularly among adolescents and young adults (1). E-cigarettes are battery-powered devices that release an inhalable aerosol by heating a liquid that contains nicotine, flavourings, and other chemical additives (2). They have become one of the most commonly used nicotine products, especially in high-income countries (1). In the United States, e-cigarettes were the second most widely used nicotine product during 2020–2021 (3). Similar trends were observed in the Middle East, particularly among young adults, where studies reported a prevalence of approximately 23% among university students in the United Arab Emirates (4) and up to 51.4% in Saudi Arabia (5).

Adolescence is a critical period for the initiation of nicotine use, and behaviours established during this stage often persist into adulthood (6). Consequently, increasing e-cigarette use among young people raises concerns about nicotine dependence, the normalisation of smoking, and subsequent transition to combustible tobacco products (7).

Several factors have contributed to the popularity of e-cigarettes, including the widespread perception that they are safer than conventional cigarettes and may aid smoking cessation (2, 8). However, this perception is misleading, as e-cigarettes expose users to nicotine as well as harmful chemicals and metals, sometimes at levels comparable to or exceeding those in traditional tobacco products (9). Nicotine exposure during adolescence is especially harmful, as it can lead to lasting cognitive and behavioral effects (6). In addition, e-cigarette use can adversely affect respiratory health, and has been associated with an increased risk of chronic conditions, including cardiovascular disease and certain cancers (10–12).

In the Middle East, e-cigarette use reflects global patterns but is shaped by regional factors such as regulatory differences, cultural norms, and varying levels of public awareness. Prevalence among university students varies widely across countries, and in many contexts, e-cigarettes are perceived as more socially acceptable than traditional smoking (13). Social media, peer influence, accessibility, and curiosity further contribute to their growing popularity among young individuals (14–17).

Within Kuwait, research examining e-cigarette use remains limited despite growing public health interest. Available studies suggest considerable use among university students, although findings are often based on specific groups and may not reflect the wider population (18, 19). Existing evidence suggests that e-cigarette use is associated with demographic and psychosocial factors, including gender, socioeconomic status, peer influence, and exposure to advertising, while greater awareness of health risks is associated with lower use (15–17).

Although studies from several countries have examined e-cigarette use among university students, important knowledge gaps remain in Kuwait. There is also limited understanding of the role of information sources, such as social media and peer networks, in shaping perceptions and behaviours. Addressing these gaps would provide evidence to guide prevention strategies and inform future tobacco control policies in Kuwait. This study aimed to assess the prevalence and patterns of e-cigarette use among students in Kuwait and to identify associated factors, including demographic characteristics, knowledge, and attitudes towards its use.

## Materials and methods

### Design

A cross-sectional study design was employed to assess the prevalence of e-cigarette use and associated factors, with data collected between November 2025 and February 2026. Eligible participants included undergraduate and postgraduate students aged 18 years or older who were enrolled in any of the randomly selected colleges or educational institutions in Kuwait, including students enrolled in diploma, bachelor’s, and postgraduate programs and were willing to provide informed consent. Responses were considered incomplete if one or more questionnaire items were unanswered and were excluded. Duplicate submissions were prevented by restricting the Google Forms questionnaire to one response per authenticated account. Following ethical approval, official letters were sent to the randomly selected institutions to facilitate data collection. Upon institutional approval, the study questionnaire was distributed to all enrolled students via email and Microsoft Teams, and advertisements were posted in the building, each including a QR code linking to the study survey. Reminder messages were sent to enhance participation rates.

### Setting

The study was conducted across universities, colleges, and vocational institutions in Kuwait, encompassing three main sectors: public universities, private universities, and colleges under the Public Authority for Applied Education and Training (PAAET). These sectors represent the primary educational pathways in the country and collectively capture the diversity of Kuwait’s postsecondary student population.

### Sample

A stratified one-stage cluster sampling approach was used to enhance representativeness across Kuwait’s major universities, colleges, and vocational institutions while maintaining logistical feasibility. Institutions were initially stratified into three categories: public universities, private universities, and PAAET-affiliated institutions. From each stratum, two institutions were randomly selected (total *n* = 6). The College of Public Health at Kuwait University was excluded from the sampling frame as it was used for pilot testing. All students affiliated with the selected six institutions were invited to participate. Informed consent was obtained electronically prior to participants’ access to the questionnaire. This approach was adopted to ensure representation across key educational sectors while maintaining feasibility for large-scale data collection. The included institutions were Kuwait University College of Business Administration, Abdullah Al Salem University College of Engineering and Energy, Kuwait International Law School, Gulf University for Science and Technology, PAAET Industrial Institute, and PAAET College of Technological Studies. The sampling and participant flow is summarised in Figure 1.

**Figure 1:**
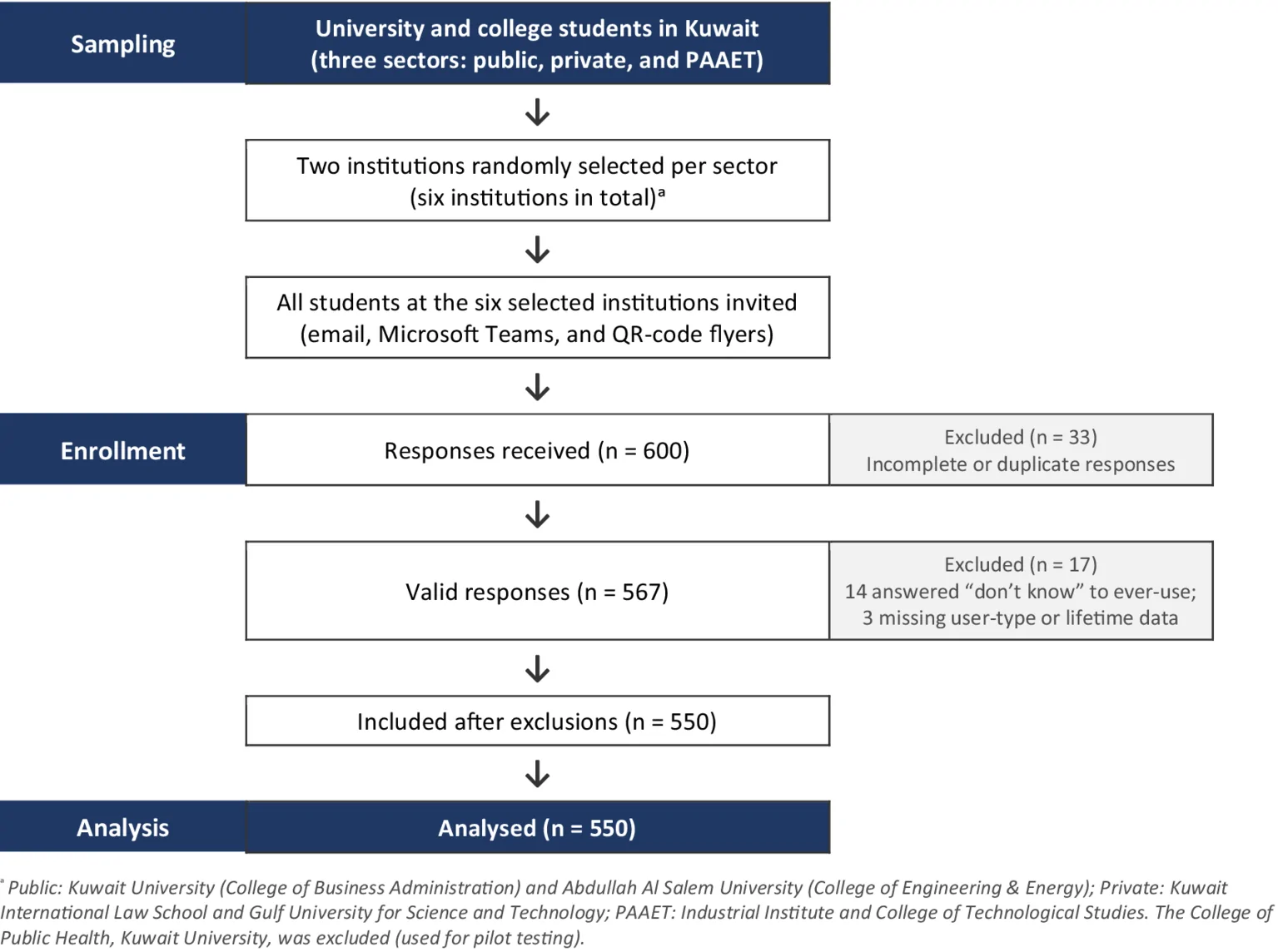
Sampling and participant flow.

The sample size was estimated to determine the required number of student participants. Assuming a 95% confidence level, a 5% margin of error, and a response distribution of 50% to maximi**s**e variability, the minimum required sample size was 384 participants. To account for the one-stage cluster sampling design, a design effect of 1.5 was applied, increasing the target to approximately 576; a further 15% allowance for incomplete responses raised the planned sample size to approximately 665.

### Questionnaire, measures, and data collection

The questionnaire was developed and adapted based on previously published literature (20, 21). A pilot test was conducted using both Arabic and English versions of the questionnaire with a sample of 20 students from the Kuwait University College of Public Health. The pilot aimed to assess comprehension of terminology and questions, evaluate the clarity and acceptability of the language, and identify any implementation challenges. Response time (in minutes) and participants’ general feedback were recorded. Based on the pilot findings, necessary revisions were made prior to administering the final version of the questionnaire across universities, colleges, and vocational institutions in Kuwait.

The final questionnaire consisted of four sections: (1) demographic and lifestyle characteristics; (2) knowledge and perceptions of e-cigarettes; (3) attitudes and beliefs toward e-cigarette use; and (4) patterns of e-cigarette use (practices), including user type and frequency of use (Supplementary material). The questionnaire was administered in both English and Arabic using Google Forms. All eligible students within the selected educational institutions were invited to participate. Data were collected using a structured, self-administered electronic questionnaire distributed via institutional student email lists and Microsoft Teams accounts. Study flyers with a QR code linking directly to the survey were also distributed to enhance participation. No personally identifiable information was collected. Participants were given the option to request a summary of the study findings. Operational definitions were provided to participants within the questionnaire. A current smoker was defined as a participant who had smoked 100 or more cigarettes in their lifetime and currently smoked every day or on some days, and a current e-cigarette user as one who had used an e-cigarette for at least 30 days, including the preceding 7 days. Regular exercise was defined as accumulating at least 150 min per week of moderate-intensity activity (e.g., brisk walking, swimming, or household or yard work) or at least 75 min per week of vigorous-intensity activity (e.g., running, cycling, football, or skipping rope). Second-hand smoking was described as the inhalation, by nearby non-smokers, of aerosol exhaled by an e-cigarette user. These questionnaire-based definitions applied to the descriptive smoking and lifestyle variables. The analytic outcome, e-cigarette use, was defined as ever-use (any lifetime use): “e-cigarette users” (ever-users) were participants who reported using an e-cigarette at least once, and “non-users” were those who reported never using one. E-cigarette use prevalence refers to ever-use (any lifetime use), and current daily use to self-reported daily use at the time of the survey. Knowledge was assessed with 22 items (Q17–Q38); each item was scored as correct (1) or incorrect (0), and the items were summed to a total knowledge score ranging from 0 to 22, with higher scores indicating greater knowledge of e-cigarettes. Attitudes were assessed with 23 items (Q40–Q62) rated on a five-point Likert scale (1 = strongly disagree to 5 = strongly agree); four items expressing support for restricting e-cigarettes (Q52–Q55: government regulation, not selling to minors, banning in public and workplace settings, and banning in Kuwait) were reverse-scored by subtracting the original response from 6 (so that 1 becomes 5, 2 becomes 4, and 3 is unchanged), because agreement with these statements reflects a less-accepting stance toward e-cigarettes; this ensured that higher total scores consistently indicated more accepting attitudes toward e-cigarette use, and the items were summed to a total attitude score ranging from 23 to 115. Total knowledge and attitude scores were classified using the modified Bloom’s cut-off points (Bloom, 1956): ≥80% of the maximum score was considered good (positive attitude), 60–79% was considered moderate (neutral), and <60% was considered poor (negative attitude). Both scales showed acceptable internal consistency (Cronbach’s α = 0.84 for knowledge and 0.76 for attitude).

### Statistical analysis

All analyses were performed using IBM SPSS Statistics (version 29). Categorical variables were summarised as frequencies and percentages, and continuous variables (age, and knowledge and attitude scores) as means and standard deviations (SD). Internal consistency of the knowledge and attitude scales was assessed using Cronbach’s α, and total knowledge and attitude scores were classified using the modified Bloom’s cut-off points. Bivariate associations between categorical variables (gender, age group, university type, and friends’ e-cigarette use) and e-cigarette use were examined using Pearson’s chi-square test of association. Differences in mean knowledge and attitude scores between groups (e-cigarette users versus non-users, and males versus females) were tested using independent-samples t-tests, with Cohen’s d reported as the effect-size measure. Differences in knowledge and attitude scores across academic years and university types were assessed using one-way analysis of variance (ANOVA); homogeneity of variances was evaluated with Levene’s test, and where this assumption was violated, the Welch test was reported together with Games–Howell post-hoc comparisons, with the Brown–Forsythe test used as a robustness check. Associations among knowledge, attitude, and age were examined using Pearson correlation coefficients.

Factors associated with e-cigarette use were examined using binary logistic regression. Unadjusted (crude) odds ratios (ORs) were obtained from univariable logistic regressions for each candidate predictor. A multivariable logistic regression model was then fitted, entering all predictors (gender, age group, university type, knowledge score, attitude score, and friends’ e-cigarette use) simultaneously, so that each adjusted odds ratio (AOR) is adjusted for all other variables in the model; predictors were selected *a priori* on the basis of the study objectives and their bivariate association with e-cigarette use. Crude and adjusted ORs are reported with 95% confidence intervals (CIs). Model performance was assessed using the Nagelkerke *R*^2^, the Hosmer–Lemeshow goodness-of-fit test, and the overall classification accuracy. A two-sided *p*-value < 0.05 was considered statistically significant.

### Ethical considerations

Ethical approval was obtained from the Kuwait University Health Sciences Centre Ethical Committee (HSCEC) (Approval No. VDR/EC 107 on 28 April 2025). Participation was voluntary, and all responses were anonymous. All data were stored on a secure drive accessible only to the research team.

## Results

### Participant characteristics

Six hundred responses were collected, 33 incomplete or duplicate questionnaires were excluded, leaving 567 valid responses. A further 14 participants who answered “do not know” to ever-use and 3 ever-users with missing user-type or lifetime data were excluded, yielding a final analytic sample of 550. Because the online survey did not enforce skip logic, use-specific responses from participants who reported never using an e-cigarette were recoded as non-use. Two hundred and seventy-seven students were E-cigarette users (ever-users) (50.4%) compared to 273 non-users (49.6%).

A total of 550 students were included in the analysis, of whom 57.3% were males. The mean age was 20.89 years (SD = 2.86), with ages ranging from 18 to 31 years. Of the sample, 41.1% were enrolled at PAAET, 30.5% at Kuwait University, and 28.4% at private universities. Among the students, 38.2% were in their second academic year, while 23.6 and 23.3% were in their first and third years, respectively. The largest proportion of students (22.5%) lived in Ahmadi governorate, followed by Hawalli governorate (21.6%). The results showed that 43.1% of the students rated their health as very good. Regarding the social environment, 53.6% indicated that their friends did not use e-cigarettes. Additionally, 48.7% of the participants reported spending 2 to 5 h per day with their friends. Moreover, most students (58.2%) indicated that they never currently smoke, while 24.0% of them are currently smoking e-cigarettes (Table 1).

**Table 1:** Participant characteristics (*N* = 550).

Variable	*n*	(%)
Age (years) Mean ± SD	550	20.89 ± 2.86
Age group		
Young ≤20	312	56.70%
Older ≥21	238	43.30%
Gender		
Male	315	57.30%
Female	235	42.70%
University type		
Public (Kuwait University)	168	30.50%
Private University	156	28.40%
PAAET	226	41.10%
Academic year		
First year	130	23.60%
Second year	210	38.20%
Third year	128	23.30%
Fourth Year+	73	13.30%
Postgraduate	9	1.60%
Governorate		
Capital (Al-Asimah)	110	20.00%
Hawalli	119	21.60%
Farwaniya	73	13.30%
Ahmadi	124	22.50%
Jahra	57	10.40%
Mubarak Al-Kabeer	67	12.20%
Self-rated health		
Excellent	188	34.20%
Very Good	237	43.10%
Good	91	16.50%
Fair	33	6.00%
Poor	1	0.20%
Exercise regularly		
Yes	248	45.1%
No	302	54.9%
Coffee consumption		
None	144	26.2%
1–3 cups/week	163	29.6%
4–6 cups/week	66	12.0%
1 cup daily	103	18.7%
>1 cup daily	74	13.5%
Smokers in Household (mean ± SD)	550	1.67 ± 1.72
Father smokes		
Yes	222	40.4%
No	328	59.6%
Mother smokes		
Yes	24	4.4%
No	526	95.6%
Friend smokes e-cigs		
Yes	255	46.40%
No	295	53.60%
Time with friends		
< 2 h/day	182	33.10%
2–5 h/day	268	48.70%
> 5 h/day	100	18.20%
Smoking status (multiple response)		
Never smoked	320	58.2%
Former smoker (quit >1 month)	47	8.5%
Current–traditional cigarettes	94	17.1%
Current–e-cigarettes	132	24.0%
Current–shisha/waterpipe	22	4.0%

Smoking status was a multiple-response item; participants could select more than one category, so percentages need not sum to 100%. Use-specific responses from non-users (current-use frequency, flavoured use, and current e-cigarette smoking) were recoded to “no use” for consistency.

### Patterns of e-cigarette use (prevalence and patterns of use)

As demonstrated in Table 2, among ever-users, 48.0% were current users, and 61.0% reported using e-cigarettes more than 50 times, while 27.4% reported using them 1–10 times during their lifetime. Of the total students, 22.5% reported daily current use and 32.4% used flavoured e-cigarettes.

**Table 2:** Patterns of e-cigarette use (prevalence and patterns of use).

Variable	*n*	(%)
Ever used e-cigarette (Q63)		
YES—ever used	277	**50.40%**
NO—never used	273	49.60%
User type (Q64)		
Current user	133	48.00%
Former user	144	52.00%
Current use frequency (Q66)		
Daily	124	22.50%
A few days/week	6	1.10%
Some days/month	1	0.20%
Rarely	33	6.00%
Not at all	386	70.20%
Lifetime use times (Q65)		
1–10 times	76	27.40%
11–20 times	18	6.50%
21–50 times	14	5.10%
> 50 times	169	61.00%
Flavoured E-Cig use (Q67)		
Yes (flavoured)	178	32.40%
No (unflavoured)	372	67.60%

### Knowledge and attitudes

Table 3 shows that the mean knowledge score was 9.23/22 (42%), corresponding to a poor level of knowledge by Bloom’s criteria (<60%). The mean attitude score was 54.38/115 (47%), which also fell below the 60% threshold; because higher scores denote greater acceptance of e-cigarette use, this indicates that students held, on average, relatively unfavourable (non-accepting) attitudes toward e-cigarette use. The results showed that e-cigarette users had a higher mean in knowledge scores than non-users. Additionally, males reported higher mean attitude scores (56.36) towards e-cigarettes compared to females (51.73). Neither knowledge nor attitude scores differed significantly across academic years. Knowledge scores differed marginally across university types (Welch’s *F*(2, 344) = 3.17, *p* = 0.043; Brown–Forsythe *p* = 0.087), with private-university students scoring highest (9.66 ± 2.61), followed by Kuwait University (9.27 ± 3.86) and PAAET students (8.91 ± 3.18); given the unequal variances (Levene’s *p* < 0.001), the Welch test is reported. Post-hoc comparisons (Games–Howell) showed this difference was significant only between private-university and PAAET students (mean difference = 0.75, 95% CI 0.05–1.45; *p* = 0.032), with private students more knowledgeable. Attitude scores did not differ significantly by university type (*F*(2, 547) = 2.67, *p* = 0.070).

**Table 3:** Knowledge and attitude scores—descriptive statistics.

Category	*n*	Knowledge scores mean ± SD	Attitude scores mean ± SD
All respondents	**550**	9.23 ± 3.27	54.38 ± 11.85
Non-user	273	8.33 ± 3.62	50.06 ± 10.22
User	277	10.12 ± 2.59	58.64 ± 11.83
Male	315	9.80 ± 3.05	56.36 ± 12.14
Female	235	8.47 ± 3.39	51.73 ± 10.94
First	130	9.35 ± 3.47	54.18 ± 11.35
Second	210	9.29 ± 3.07	54.09 ± 11.44
Third	128	8.81 ± 2.97	55.23 ± 12.16
Fourth+	73	9.44 ± 3.70	53.82 ± 12.97
Postgraduate	9	10.56 ± 4.88	56.56 ± 16.12

According to Table 4, the t-test indicates that e-cigarette users reported significantly higher levels of knowledge than non-users. Moreover, males have significantly more positive attitudes towards e-cigarettes than females, with *p*-values < 0.001 for both.

**Table 4:** Comparison of knowledge and attitude scores by e-cigarette use and gender (independent-samples *t*-tests).

Comparison	Group	*n*	Mean ± SD	*t* (*df*)	*p*	Cohen’s *d*
Knowledge, by e-cigarette use	Non-user	273	8.33 ± 3.62	−6.66 (548)	<0.001	−0.57
	User	277	10.12 ± 2.59			
Knowledge, by gender	Male	315	9.80 ± 3.05	4.84 (548)	<0.001	0.42
	Female	235	8.47 ± 3.39			
Attitude, by e-cigarette use	Non-user	273	50.06 ± 10.22	−9.09 (548)	<0.001	−0.78
	User	277	58.64 ± 11.83			
Attitude, by gender	Male	315	56.36 ± 12.14	4.61 (548)	<0.001	0.40
	Female	235	51.73 ± 10.94			

### Factors associated with e-cigarette use

In the chi-square test, gender, friends’ e-cigarette use, and university type were significantly associated with e-cigarette use (*p* < 0.001). Males were significantly more likely to use than females. Among participants whose friends used e-cigarettes, 76.9% (196/255) were themselves e-cigarette users, compared with 27.5% (81/295) among those whose friends did not. Private university students had a higher proportion of e-cigarette users (55.1%) than public university students (33.9%). PAAET students showed the highest proportion of e-cigarette use (59.3%). In addition, there was a significant association between age group and e-cigs (*p* = 0.002). Older students (≥21 years) were more likely to use e-cigarettes compared to younger students (≤20 years) (Table 5).

**Table 5:** Factors associated with e-cigarette use (crude and adjusted odds ratios).

Variable	Users (*n*)	Non-users (*n*)	Crude OR (95% CI)	*p*	Adjusted OR (95% CI)	*p*
Gender						
Female	58	177	1.00 (reference)		1.00 (reference)	
Male	219	96	6.96 (4.75–10.20)	<0.001	3.70 (2.28–6.02)	<0.001
Age group						
Young ≤20	139	173	1.00 (reference)		1.00 (reference)	
Older ≥21	138	100	1.72 (1.22–2.42)	0.002	1.45 (0.93–2.27)	0.098
University type						
Public (Kuwait University)	57	111	1.00 (reference)		1.00 (reference)	
Private	86	70	2.39 (1.53–3.75)	<0.001	2.36 (1.31–4.24)	0.004
PAAET	134	92	2.84 (1.87–4.30)	<0.001	2.53 (1.46–4.41)	0.001
Friends’ e-cigarette use						
No	81	214	1.00 (reference)		1.00 (reference)	
Yes	196	59	8.78 (5.96–12.93)	<0.001	4.53 (2.90–7.08)	<0.001
Knowledge score (per point)	—	—	1.198 (1.132–1.269)	<0.001	1.164 (1.079–1.254)	<0.001
Attitude score (per point)	—	—	1.072 (1.054–1.091)	<0.001	1.068 (1.046–1.091)	<0.001

*N* = 550 (277 e-cigarette users [ever-users] and 273 non-users). Counts show users vs non-users per category; all categorical associations were significant on chi-square (gender, friends, and university type *p* < 0.001; age group *p* = 0.002). Crude ORs are unadjusted (from univariable logistic regression); adjusted ORs are from a single multivariable model with all listed variables entered simultaneously (Nagelkerke *R*^2^ = 0.508; Hosmer–Lemeshow *p* = 0.260; 79.3% correctly classified). Knowledge and attitude ORs are per one-point increase.

Table 5 identifies the predictors of e-cigarette use in the analytic sample (*N* = 550) (Nagelkerke *R*^2^ = 0.508; Hosmer–Lemeshow *p* = 0.260). The strongest predictor was having friends who smoke e-cigarettes, followed by university type, gender (male), and higher attitude and knowledge scores. The odds of using e-cigarettes were significantly higher among students who had friends who smoked e-cigarettes (AOR = 4.53, 95% CI 2.90–7.08; *p* < 0.001). Male students had 3.70 times higher odds of e-cigarette use than female students (*p* < 0.001). Compared with public university (Kuwait University) students, those attending private universities (AOR = 2.36; *p* = 0.004) and PAAET institutions (AOR = 2.53; *p* = 0.001) had significantly higher odds of use. E-cigarette use was also significantly associated with higher attitude (AOR = 1.068; *p* < 0.001) and higher knowledge (AOR = 1.164; *p* < 0.001) scores. Age group was not an independent predictor of e-cigarette use after adjustment (AOR = 1.45; *p* = 0.098).

### Secondary analyses

Attitude scores did not differ significantly across academic years (one-way ANOVA, *F* = 0.32, *p* = 0.865), and neither did knowledge scores (*F* = 1.03, *p* = 0.393). Knowledge and attitude scores were not significantly correlated (r = 0.05, *p* = 0.23), nor was either associated with age (knowledge: r = −0.003, *p* = 0.95; attitude: r = 0.01, *p* = 0.76).

## Discussion

The study showed that among 550 university students in Kuwait, 50.4% reported ever using e-cigarettes. Nearly a quarter of the sample (22.5%) reported daily current use, and approximately one third (32.4%) used flavoured products. Overall knowledge (9.23/22, 42%) and attitude scores (54.38/115, 47%) were below Bloom’s threshold for adequacy, indicating generally poor knowledge and predominantly non-accepting attitudes toward e-cigarette use across the sample. However, e-cigarette users scored significantly higher than non-users on both knowledge and attitude measures, and males scored higher than females on both measures. Bivariate analyses identified gender, friends’ e-cigarette use, university type, and age group as significantly associated with e-cigarette use. In the multivariable model, having friends who use e-cigarettes was the strongest independent predictor (AOR = 4.53), followed by male gender (AOR = 3.70), attendance at a PAAET or private institution compared to Kuwait University (AOR = 2.53 and 2.36, respectively), and higher attitude and knowledge scores (AOR = 1.068 and 1.164).

In the present sample, 50.4% reported ever-use of e-cigarettes (of whom 48.0% were current and 52.0% former users), and 22.5% reported current daily use. Comparing like with like, the ever-use prevalence (50.4%) was higher than the ever-use and overall estimates reported among students elsewhere — for example, 15.5% ever-use in an earlier sample of Kuwait University students (22), 22% lifetime use and 22.65% overall in two systematic reviews and meta-analyses (1, 23), and 29.9% overall in Saudi Arabia (24). The current daily-use estimate (22.5%) was likewise higher than the current-use estimates reported in comparable studies, including 10.2% current use (23), 23.5% current use in the United Arab Emirates (25), and 14% current use in Qatar (26).

The difference in usage rates between this study’s results and those of the other study conducted in Kuwait (22) can be explained by the inclusion of private universities and PAEET, which showed higher rates of e-cigarette use among their students. An explanation for the high prevalence in Kuwait in comparison to global levels can be that e-cigarette smoking is high among high-income countries’ students (1).

E-cigarette use is higher in Kuwait than in some neighbouring Gulf states, likely due to more lenient regulations and lower taxes. While countries like Bahrain, Oman, and Qatar have banned e-cigarette sales, and others like Saudi Arabia and the UAE impose a 100% tax, Kuwait does not tax e-cigarettes (27, 28). A unified Gulf standard for vaping products was approved in 2025 (29), but implementation remains unclear, and Kuwait still follows older regulations. In March 2026, the Ministry of Commerce and Industry banned the sale of e-cigarettes and related accessories through delivery platforms and other digital channels (30), while in-person retail sales remain comparatively unregulated. This slower, partial approach in Kuwait contrasts with stricter policies elsewhere, supporting evidence that higher taxes reduce e-cigarette use (31, 32).

The study’s results showed that the sample had low overall knowledge, which is broadly concordant with other studies in the literature (5, 20, 33); however, direct comparison is limited by differences in study populations, settings, measurement instruments, and knowledge scoring/classification systems across these studies. The finding that e-cigarette users exhibit significantly higher knowledge scores than non-users aligns with previous research (34, 35); however, those studies differed in their target populations, knowledge instruments, and scoring systems, so this concordance should be interpreted with caution rather than as a direct equivalence. This outcome may indicate that knowledge is acquired through exposure or experience with e-cigarettes, rather than serving as a protective or preventive factor. Notably, this trend contradicts the predictions of the Health Belief Model, which posits that increased knowledge of risk should reduce use (36). Several authors have characterised this phenomenon as a ‘knowledge-attitude’ or ‘knowledge–behaviour’ paradox, in which awareness of e-cigarettes’ relative characteristics, including their perceived reduced harm compared to conventional cigarettes, appears to reinforce rather than discourage continued use (37, 38).

Regarding the attitudes towards e-cigarette use, the results showed that the sample had relatively unfavourable or non-accepting attitudes toward e-cigarettes, a result that was also shown in other studies in the literature (26, 39, 40). However, users had more permissive attitudes, which is a finding also mentioned in the literature (26, 35). As in other studies (26, 41, 42), peer influence was shown to have a statistically significant risk for e-cigarette use. Being a male was also a risk factor for e-cigarette use and having higher knowledge and more permissive attitudes towards use (1, 26, 41). Another significant finding was the higher odds of use among PAAET and private university students, which can be explained by the lower level of education among PAAET students, as it is a diploma-level institution, and by the different socioeconomic levels and cultural differences in private universities, which have more non-Kuwaiti students. Such an explanation is supported by studies, which have shown that lower educational attainment (43, 44) and diverse socioeconomic and cultural values might influence e-cigarette use (45).

### Strengths and limitations

This study has several strengths. Firstly, it uses a stratified one-stage cluster sampling approach, which ensured a diverse and representative sample across Kuwait’s public, private, and PAAET sectors. Additionally, the study utilised a pilot-tested questionnaire in both Arabic and English, demonstrating acceptable internal consistency (Cronbach’s α of 0.84 for knowledge and 0.76 for attitude) for its primary measures. Despite these strengths, several limitations should be considered when interpreting the findings. First, the cross-sectional design allows for the identification of associations but prevents the establishment of definitive causal relationships between the predictors and e-cigarette use. Second, the study relied on self-reported data collected via an electronic questionnaire, which may be subject to recall bias or social desirability bias, potentially leading students to underreport their usage or misrepresent their attitudes. Third, although the sampling approach enhanced representativeness, the distribution of the survey via email and Microsoft Teams may have introduced selection bias, as participation was limited to those who chose to respond to the digital invitation. Finally, while the study captured a range of university, college, and vocational education sectors, the results are specific to university students and may not be generalisable to the broader population of Kuwait or to adolescents not yet in higher education.

### Future studies and policy implications

The high prevalence of e-cigarette use among Kuwaiti university students, which exceeds that in several neighbouring Gulf countries, indicates a need for stronger regulatory measures. Unlike other GCC countries, Kuwait does not currently tax e-cigarettes. Given that taxation is associated with reduced use (31), implementing a substantial excise tax, informed by price-elasticity evidence, should be considered. While the forthcoming 2026 digital sales ban represents progress, more stringent regulation of in-person retail sales is likely necessary to effectively restrict access (46). Peer influence emerged as the strongest predictor of e-cigarette use. In conjunction with the knowledge-attitude paradox identified in this study, these findings suggest that conventional educational campaigns may be insufficient. Instead, Interventions should prioritise modifying peer-driven social norms, such as implementing peer-led campus initiatives. This approach is supported by emerging trial evidence indicating that peer-to-peer education can strengthen students’ cognitive resistance to e-cigarette use (47). Furthermore, it aligns with broader recommendations that policies for this population should specifically target social norms through peer-led programming (48).

For future research, longitudinal cohort studies are needed to determine whether e-cigarette use precedes traditional smoking and to monitor long-term health outcomes in this population. Qualitative research should investigate the cultural and social determinants of vaping, including the influence of social media and peer networks. Future research should directly examine the knowledge-attitude paradox, including whether harm-reduction or risk-emphasis messaging differentially influences attitudes and behaviours. Expanding research populations beyond university students to include school-aged adolescents and non-enrolled young adults would help establish national prevalence estimates and identify earlier intervention opportunities. Additionally, prospective assessment of the 2026 digital sales ban and any forthcoming taxation policies will be critical to optimising Kuwait’s tobacco control strategy.

## Conclusion

This study identifies a significant public health concern: over half of university students in Kuwait have used e-cigarettes, and nearly a quarter report daily use. This prevalence is notably higher than in several neighbouring Gulf countries, which may reflect Kuwait’s relatively permissive regulatory environment and the absence of e-cigarette-specific taxation; however, the cross-sectional design precludes confirmation of this association. A key finding is the “knowledge-attitude paradox”: users demonstrated greater knowledge than non-users, indicating that awareness may reinforce rather than deter use. Peer influence was among the strongest predictors of use. Elevated risk among male students and those attending private or PAAET institutions suggests that cultural, socioeconomic, and educational factors shape vaping behaviour, although these associations remain hypothesis-generating. Addressing this trend will require stricter national regulation aligned with GCC standards, including taxation and retail restrictions, as well as public health campaigns that extend beyond knowledge dissemination to address peer-driven norms and misconceptions regarding the safety of e-cigarettes.

## Data Availability

The datasets presented in this article are not readily available because restrictions according to the regulations of the ethical committee. Requests to access the datasets should be directed to a.alhenaidi@hotmail.com.
