# Supplementary material for: Prevalence, knowledge, attitudes, and practices related to e-cigarette use among students in Kuwait: a cross-sectional study
Source: Front Public Health. 2026 Oct 8;14:1947592. doi: 10.3389/fpubh.2026.1947592 (PMC13687304; doi:10.3389/fpubh.2026.1947592)
Supplement: Supplementary file 1 [file Data_Sheet_1.docx]

**Appendix. Study Questionnaire (Bilingual)**

**الملحق: استبيان الدراسة (نسخة ثنائية اللغة)**

*Administered in English and Arabic via Google Forms, in four sections. Knowledge items (Q17–Q38) were scored 1 (correct) / 0 (incorrect or “don’t know”); attitude items (Q40–Q62) used a five-point Likert scale, with the four restriction items (Q52–Q55) reverse-coded so higher totals indicate more accepting attitudes toward e-cigarette use.*

**Section 1 — Sociodemographic and Lifestyle Characteristics (Q1–Q16)**

**القسم الأول — الأسئلة الديموغرافية والاجتماعية**

**1.** What is your age?

ما هو عمرك؟

**2.** What is your gender?

*Male / Female*

يرجى تحديد الجنس؟

*ذكر / أنثى*

**3.** What type of university do you attend?

*Public University / Private University / The Public Authority for Applied Education and Training (PAAET)*

ما نوع الجامعة التي تدرس فيها؟

*جامعة حكومية / جامعة خاصة*

**4.** Academic Year

*First / Second / Third / Fourth or more / Higher education studies*

السنة الدراسية

*أولى / ثانية / ثالثة / رابعة أكثر / دراسات عليا*

**5.** What governorate do you live in?

*Al-Asimah (Capital) / Hawalli / Ahmadi / Farwaniya / Jahra / Mubarak Al-Kabeer*

في أي محافظة تسكن؟

*العاصمة / حولي / الأحمدي / الجهراء / الفروانية / مبارك الكبير*

**6.** In general, how would you rate your overall health?

*Excellent / Very good / Good / Fair / Poor*

بشكل عام، كيف تقيم صحتك؟

*ممتاز / جيد جداً / جيد / متوسط / سيء*

**7.** Do you currently smoke? (you can choose more than one answer) You are identified as a smoker if you have ever smoked 100 cigarettes or more in your lifetime and currently smoke every day or on some days. You are considered an e-cigarette smoker if you have used an e-cigarette for at least 30 days, including the past 7 days.

*No, I never smoked / No, I quit (more than 1 month ago) / Yes, traditional cigarettes / Yes, shisha / Yes, e-cigarettes*

هل أنت مدخن حالياً؟ (يمكنك اختيار أكثر من إجابة) يتم تصنيفك كمدخن إذا سبق لك أن دخنت 100 سيجارة أو أكثر في حياتك وتدخن حاليًا كل يوم أو في بعض الأيام. يتم تصنيفك مدخنًا للسجائر الإلكترونية إذا كنت قد استخدمت سيجارة إلكترونية لمدة 30 يومًا على الأقل، بما في ذلك الأيام السبعة الماضية.

*لا، لم أدخن أبدًا / لا، أقلعت عن التدخين (لأكثر من شهر) / نعم، أدخن السجائر التقليدية / نعم، أدخن الشيشة / نعم، أستخدم السجائر الإلكترونية*

**8.** At what age did you start smoking? (in years)

في أي عمر بدأت التدخين؟

**9.** For how many years did you smoke? (in years)

كم سنة كنت تدخن؟

**10.** Do you exercise regularly? You are considered to have regular exercise if you accumulate a minimum of 150 minutes per week through activities such as brisk walking, swimming, household or yard work, or 75 minutes per week through activities like running, cycling, playing football, or jumping rope.

*Yes / No*

هل تمارس الرياضة بانتظام؟ يُعتبر الشخص مُمارسًا للتمارين الرياضية بانتظام إذا مارس ما لا يقل عن 150 دقيقة أسبوعيًا من خلال أنشطة مثل المشي السريع والسباحة والأعمال المنزلية أو أعمال الحديقة، أو 75 دقيقة أسبوعيًا من خلال أنشطة مثل الجري وركوب الدراجات ولعب كرة القدم والقفز بالحبل

*نعم / لا*

**11.** Do you drink coffee? (One cup = 227 ml)

*No / Yes: 1–3 cups a week / Yes: 4–6 cups a week / Yes: 1 cup daily / Yes: More than 1 cup daily*

هل تشرب القهوة؟ الكوب الواحد = ٢٢٧ مل

*لا / نعم: من 1 إلى 3 أكواب أسبوعيًا / نعم: من 4 إلى 6 أكواب أسبوعيًا / نعم: كوب واحد يوميًا / نعم: أكثر من كوب يوميًا*

**12.** Number of smokers where you live:

عدد المدخنين في سكنك

**13.** Is your father a smoker?

*Yes / No*

هل والدك مُدخن؟

*نعم / لا*

**14.** Is your mother a smoker?

*Yes / No*

هل والدتك مدخنة؟

*نعم / لا*

**15.** Is one of your close friends a smoker? (you can choose more than one answer)

*No / Yes, traditional cigarettes / Yes, shisha / Yes, e-cigarettes*

هل أحد أصدقائك المقربين مدخن؟ (يمكنك اختيار أكثر من إجابة)

*نعم، الشيشة / نعم، السجائر الإلكترونية / نعم، السجائر التقليدية / لا*

**16.** How much time do you spend with your friends daily?

*Less than 2 hours / 2–5 hours / More than 5 hours*

كم من الوقت تقضيه مع أصدقائك يوميًا؟

*أقل من ساعتين / من ساعتين إلى خمس ساعات / اكثر من خمس ساعات*

**Section 2 — Knowledge and Perceptions (Q17–Q39)**

**القسم الثاني — المعرفة**

**17.** Have you ever heard about e-cigarettes?

*I don't know / No / Yes*

هل سمعتَ يومًا عن السجائر الإلكترونية من قبل؟

*لا أعرف / لا / نعم*

**18.** Are E-cigarettes addictive?

*I don't know / No / Yes*

هل السجائر الإلكترونية تُسبب الإدمان؟

*لا أعرف / لا / نعم*

**19.** Are e-cigarettes a potential cause of asthma attacks and allergies?

*I don't know / No / Yes*

هل السجائر الإلكترونية هي سبب من الأسباب المحتملة لنوبات الربو والحساسية؟

*لا أعرف / لا / نعم*

**20.** Can e-cigarettes contain nicotine?

*I don't know / No / Yes*

هل تحتوي السجائر الإلكترونية على النيكوتين؟

*لا أعرف / لا / نعم*

**21.** Is the health risk of e-cigarettes the same as that of traditional cigarettes?

*I don't know / No / Yes*

هل الأخطار الصحية من استخدام السجائر الإلكترونية هي ذات الأخطار لاستخدام للسجائر العادية؟

*لا أعرف / لا / نعم*

**22.** Do e-cigarettes have the same chemicals as traditional cigarettes?

*I don't know / No / Yes*

هل تحتوي السجائر الإلكترونية على نفس المواد الكيميائية الموجودة في السجائر العادية

*لا أعرف / لا / نعم*

**23.** Are you aware of any regulations by the government on e-cigarettes?

*I don't know / No / Yes*

هل أنت على علم بأي قانون صادر من الحكومة بشأن السجائر الإلكترونية؟

*لا أعرف / لا / نعم*

**24.** Are e-cigarettes less harmful to health than traditional cigarettes?

*I don't know / No / Yes*

هل استخدام السجائر الإلكترونية أقل ضرراً على الصحة من استخدام السجائر العادية؟

*لا أعرف / لا / نعم*

**25.** Are e-cigarettes harmful to health?

*I don't know / No / Yes*

هل السجائر الإلكترونية ضارة بالصحة؟

*لا أعرف / لا / نعم*

**26.** Can e-cigarettes be used in non-smoking areas?

*I don't know / No / Yes*

هل يمكن استخدام الإلكترونية في الأماكن التي يمنع التدخين فيها؟

*لا أعرف / لا / نعم*

**27.** Is e-cigarette vapor dangerous to babies and kids?

*I don't know / No / Yes*

هل يعد دخان السجائر الإلكترونية خطيراً على الرضع والأطفال؟

*لا أعرف / لا / نعم*

**28.** Are e-cigarettes associated with bladder cancer?

*I don't know / No / Yes*

هل استخدام السجائر الإلكترونية مرتبط بسرطان المثانة؟

*لا أعرف / لا / نعم*

**29.** Has the American Food and Drug Administration (FDA) approved e-cigarettes?

*I don't know / No / Yes*

هل السجائر الإلكترونية معتمدة من إدارة الغذاء والدواء الأمريكية؟

*لا أعرف / لا / نعم*

**30.** Are some flavors of e-cigarettes more harmful than others?

*I don't know / No / Yes*

هل تعتبر بعض نكهات السجائر الإلكترونية أكثر ضررًا من غيرها من النكهات؟

*لا أعرف / لا / نعم*

**31.** Can accidentally swallowing the liquid in e-cigarettes cause potentially fatal poisoning?

*I don't know / No / Yes*

هل ابتلاع السائل الموجود في السجائر الإلكترونية عن طريق الخطأ قد يسبب تسممًا قد يكون مميتًا؟

*لا أعرف / لا / نعم*

**32.** Are e-cigarettes not associated with lung cancer?

*I don't know / No / Yes*

هل السجائر الإلكترونية غير مرتبطة بسرطان الرئة؟

*لا أعرف / لا / نعم*

**33.** Can e-cigarettes negatively affect the functioning of the heart?

*I don't know / No / Yes*

هل السجائر الإلكترونية تؤثر سلبًا على وظائف القلب؟

*لا أعرف / لا / نعم*

**34.** Are e-cigarettes associated with decreased male fertility?

*I don't know / No / Yes*

هل ترتبط السجائر الإلكترونية بانخفاض خصوبة الرجال؟

*لا أعرف / لا / نعم*

**35.** Do e-cigarettes contribute to secondhand smoking? (refers to the inhalation of smoke released from a smoking device, such as the smoke that may be exhaled through the mouth or nose of a e-cigarette user from nearby non-smokers)

*I don't know / No / Yes*

هل تُسهم السجائر الإلكترونية في التدخين السلبي؟ (أي استنشاق الدخان المنبعث من جهاز التدخين، مثل الدخان الذي قد يُخرج من فم أو أنف مستخدم السجائر الإلكترونية، من قبل المقربين من غير المدخنين)

*لا أعرف / لا / نعم*

**36.** Are e-cigarettes suitable for pregnant women?

*I don't know / No / Yes*

هل السجائر الإلكترونية مناسبة للحوامل؟

*لا أعرف / لا / نعم*

**37.** Do e-cigarettes in Kuwait contain nicotine?

*I don't know / No / Yes*

هل تحتوي السجائر الإلكترونية في الكويت على النيكوتين؟

*لا أعرف / لا / نعم*

**38.** Do e-cigarettes contain other harmful substances besides nicotine?

*I don't know / No / Yes*

هل تحتوي السجائر الإلكترونية على مواد ضارة أخرى غير النيكوتين؟

*لا أعرف / لا / نعم*

**39.** Have you heard about e-cigarettes? If yes, from which source did you get your information? Check all that apply. (If ‚Äúother‚Äù, please describe it below)

*Social media / Online advertising / Television / Radio / Billboards / Newspapers or magazines / Friends or family / School or university / Other — [descriptive item; not scored]*

هل سمعتَ عن السجائر الإلكترونية؟ إذا نعم، فمن أي مصدر حصلتَ على معلوماتك؟

*وسائل التواصل الاجتماعي (تويتر، سناب شات، إنستغرام، إلخ.) / الإعلان عبر الإنترنت / الإعلان التلفزيوني / الإعلان الإذاعي / اللوحات الإعلانية و/أو اللافتات العامة / الصحف أو المجلات / الأصدقاء/العائلة. / أخرى (حدد _______ )*

**Section 3 — Attitudes and Beliefs (Q40–Q62)**

**القسم الثالث — المعتقدات والتوجهات**

**40.** Using e-cigarettes is fun.

*Strongly agree / Agree / Not sure / Disagree / Strongly disagree*

استخدام السجائر الإلكترونية أمر ممتع

*أوافق بشدة / أوافق / لست متأكداً / لا أوافق / لا أوافق نهائياً*

**41.** Using e-cigarettes is considered more socially acceptable than smoking traditional cigarettes.

*Strongly agree / Agree / Not sure / Disagree / Strongly disagree*

يُعتبر استخدام السجائر الإلكترونية أكثر قبولاً اجتماعياً من تدخين السجائر التقليدية

*أوافق بشدة / أوافق / لست متأكداً / لا أوافق / لا أوافق نهائياً*

**42.** E-cigarettes relieve one's stress.

*Strongly agree / Agree / Not sure / Disagree / Strongly disagree*

السجائر الإلكترونية تُخفف من حدة التوتر

*أوافق بشدة / أوافق / لست متأكداً / لا أوافق / لا أوافق نهائياً*

**43.** E-cigarettes help to cut down tobacco smoking.

*Strongly agree / Agree / Not sure / Disagree / Strongly disagree*

تُساعد السجائر الإلكترونية في التقليل من تدخين التبغ

*أوافق بشدة / أوافق / لست متأكداً / لا أوافق / لا أوافق نهائياً*

**44.** E-cigarettes could serve as a beneficial method for aiding individuals in quitting smoking.

*Strongly agree / Agree / Not sure / Disagree / Strongly disagree*

يمكن أن تُشكل السجائر الإلكترونية وسيلة مفيدة لمساعدة الأفراد على الإقلاع عن التدخين

*أوافق بشدة / أوافق / لست متأكداً / لا أوافق / لا أوافق نهائياً*

**45.** E-cigarettes are safer compared to traditional cigarettes.

*Strongly agree / Agree / Not sure / Disagree / Strongly disagree*

السجائر الإلكترونية أكثر أمانًا مقارنةً بالسجائر التقليدية

*أوافق بشدة / أوافق / لست متأكداً / لا أوافق / لا أوافق نهائياً*

**46.** People who use e-cigarettes are not regarded as traditional smokers.

*Strongly agree / Agree / Not sure / Disagree / Strongly disagree*

لا يُعتبر مستخدمو السجائر الإلكترونية مدخنين تقليديين

*أوافق بشدة / أوافق / لست متأكداً / لا أوافق / لا أوافق نهائياً*

**47.** E-cigarettes make someone look stylish.

*Strongly agree / Agree / Not sure / Disagree / Strongly disagree*

السجائر الإلكترونية تجعل الشخص يبدو أنيقاً

*أوافق بشدة / أوافق / لست متأكداً / لا أوافق / لا أوافق نهائياً*

**48.** E-cigarettes improve one's image.

*Strongly agree / Agree / Not sure / Disagree / Strongly disagree*

تُحسّن السجائر الإلكترونية صورة الشخص

*أوافق بشدة / أوافق / لست متأكداً / لا أوافق / لا أوافق نهائياً*

**49.** E-cigarettes cost less money than traditional cigarettes.

*Strongly agree / Agree / Not sure / Disagree / Strongly disagree*

السجائر الإلكترونية أقل تكلفة من السجائر التقليدية

*أوافق بشدة / أوافق / لست متأكداً / لا أوافق / لا أوافق نهائياً*

**50.** E-cigarettes are easier to get than traditional cigarettes.

*Strongly agree / Agree / Not sure / Disagree / Strongly disagree*

السجائر الإلكترونية أسهل في الحصول عليها من السجائر التقليدية

*أوافق بشدة / أوافق / لست متأكداً / لا أوافق / لا أوافق نهائياً*

**51.** E-cigarettes are more pleasurable than traditional cigarettes.

*Strongly agree / Agree / Not sure / Disagree / Strongly disagree*

السجائر الإلكترونية أكثر متعة من السجائر التقليدية

*أوافق بشدة / أوافق / لست متأكداً / لا أوافق / لا أوافق نهائياً*

**52.** The government should regulate the use of e-cigarettes.

*Strongly agree / Agree / Not sure / Disagree / Strongly disagree*

يجب على الحكومة تنظيم استخدام السجائر الإلكترونية

*أوافق بشدة / أوافق / لست متأكداً / لا أوافق / لا أوافق نهائياً*

**53.** E-cigarettes should not be sold to minors.

*Strongly agree / Agree / Not sure / Disagree / Strongly disagree*

يجب عدم بيع السجائر الإلكترونية للقاصرين

*أوافق بشدة / أوافق / لست متأكداً / لا أوافق / لا أوافق نهائياً*

**54.** The use of e-cigarettes should be banned in both workplace and public settings.

*Strongly agree / Agree / Not sure / Disagree / Strongly disagree*

يجب حظر استخدام السجائر الإلكترونية في أماكن العمل والأماكن العامة

*أوافق بشدة / أوافق / لست متأكداً / لا أوافق / لا أوافق نهائياً*

**55.** E-cigarettes should be banned in Kuwait.

*Strongly agree / Agree / Not sure / Disagree / Strongly disagree*

يجب حظر السجائر الإلكترونية في الكويت

*أوافق بشدة / أوافق / لست متأكداً / لا أوافق / لا أوافق نهائياً*

**56.** E-cigarette use is regarded as a sign of sophistication and civilization.

*Strongly agree / Agree / Not sure / Disagree / Strongly disagree*

يُعتبر استخدام السجائر الإلكترونية دليلاً على الرقي والتحضر

*أوافق بشدة / أوافق / لست متأكداً / لا أوافق / لا أوافق نهائياً*

**57.** The existence of various e-cigarette flavors plays a role in promoting the spread of this trend.

*Strongly agree / Agree / Not sure / Disagree / Strongly disagree*

يلعب وجود نكهات مختلفة للسجائر الإلكترونية دورًا في تعزيز انتشار هذا التوجه

*أوافق بشدة / أوافق / لست متأكداً / لا أوافق / لا أوافق نهائياً*

**58.** E-cigarette use can lead to the initiation of traditional cigarette smoking.

*Strongly agree / Agree / Not sure / Disagree / Strongly disagree*

قد يؤدي استخدام السجائر الإلكترونية إلى بدء تدخين السجائر التقليدية

*أوافق بشدة / أوافق / لست متأكداً / لا أوافق / لا أوافق نهائياً*

**59.** E-cigarette advertisements make using e-cigarettes look cool.

*Strongly agree / Agree / Not sure / Disagree / Strongly disagree*

إعلانات السجائر الإلكترونية تجعل استخدامها يبدو جذابًا

*أوافق بشدة / أوافق / لست متأكداً / لا أوافق / لا أوافق نهائياً*

**60.** E-cigarettes have a problem-solving effect.

*Strongly agree / Agree / Not sure / Disagree / Strongly disagree*

للسيجارة الإلكترونية تأثير في حل المشكلات

*أوافق بشدة / أوافق / لست متأكداً / لا أوافق / لا أوافق نهائياً*

**61.** E-cigarettes enhance one's performance.

*Strongly agree / Agree / Not sure / Disagree / Strongly disagree*

السجائر الإلكترونية تُحسّن أداء الشخص

*أوافق بشدة / أوافق / لست متأكداً / لا أوافق / لا أوافق نهائياً*

**62.** E-cigarettes increase one's concentration.

*Strongly agree / Agree / Not sure / Disagree / Strongly disagree*

السجائر الإلكترونية تزيد من التركيز

*أوافق بشدة / أوافق / لست متأكداً / لا أوافق / لا أوافق نهائياً*

**Section 4 — E-cigarette Practices (Q63–Q67)**

**القسم الرابع — الممارسات المتعلقة بالسجائر الإلكترونية**

**63.** Have you ever used an electronic cigarette, even if it's just one time in your entire life?

*Yes / No / I don't know. If no or don't know, the following questions do not apply.*

هل سبق لك استخدام سيجارة إلكترونية، حتى لو لمرة واحدة فقط في حياتك؟

*نعم / لا / لا أعرف/لست متأكدًا*

**64.** If yes (you used an electronic cigarette, even if it's just one time in your entire life), are you a:

*Current user / Former user*

إذا كانت الإجابة نعم، فهل أنت:

*مستخدم حالي؟ / مستخدم سابق (جربته من قبل)*

**65.** How many times in total do you think you have used an e-cigarette during your lifetime? (If you did not use electronic cigarette before, go to the next question)

*1 to 10 times / 11 to 20 times / 21 to 50 times / More than 50 times*

كم عدد المرات في حياتك التي استخدمت فيها سيجارة إلكترونية؟

*من 1 إلى 10 / من 11 إلى 20 / من 21 إلى 50 / أكثر من 50 مرة*

**66.** Currently, how frequently do you use e-cigarettes?

*Every day / Every few days / Some days / Rarely / I don't use them at all*

حالياً، ما مدى تكرار استخدامك للسجائر الإلكترونية حالياً؟

*كل يوم / كل بضعة أيام/ بعض أيام / نادراً / لا أستخدمها على الإطلاق*

**67.** Were any of the electronic cigarettes that you used in the past 30 days flavored to taste like menthol, mint, clove, spice, candy, fruit, chocolate, or other sweets?

*Yes / No*

هل كانت أي من السجائر الإلكترونية التي استخدمتها خلال الثلاثين يومًا الماضية لها نكهة مثل المنثول، أو النعناع، أو القرنفل، أو التوابل، أو الحلوى، أو الفاكهة، أو الشوكولاتة، أو غيرها من الحلويات؟

*نعم / لا /*
